# Physical inactivity by tail suspension alters markers of metabolism, structure, and autophagy of the mouse heart

**DOI:** 10.14814/phy2.15574

**Published:** 2023-01-25

**Authors:** Ana Victoria Rojo‐García, Mathias Vanmunster, Alexander Pacolet, Frank Suhr

**Affiliations:** ^1^ Department of Movement Sciences Exercise Physiology Research Group, KU Leuven Leuven Belgium

**Keywords:** cardiomyocytes, heart, mouse, physical inactivity, tail suspension

## Abstract

Sedentary behavior has become ingrained in our society and has been linked to cardiovascular diseases. Physical inactivity is the main characteristic of sedentary behavior. However, its impact on cardiovascular disease is not clear. Therefore, we investigated the effect of physical inactivity in an established mouse model on gene clusters associated with cardiac fibrosis, electrophysiology, cell regeneration, and tissue degradation/turnover. We investigated a sedentary group (CTR, *n* = 10) versus a tail suspension group (TS, *n* = 11) that caused hindlimb unloading and consequently physical inactivity. Through histological, protein content, and transcript analysis approaches, we found that cardiac fibrosis‐related genes partly change, with significant TS‐associated increases in *Tgfb1*, but without changes in *Col1a1* and *Fn1*. These changes are not translated into fibrosis at tissue level. We further detected TS‐mediated increases in protein degradation‐ (*Trim63*, *p* < 0.001; *Fbxo32*, *p* = 0.0947 as well as in biosynthesis‐related [*P70s6kb1*, *p* < 0.01]). Corroborating these results, we found increased expression of autophagy markers such as *Atg7* (*p* < 0.01) and *ULK1* (*p* < 0.05). Two cardiomyocyte regeneration‐ and sarcomerogenesis‐related genes, *Yap* (*p* = 0.0535) and *Srf* (*p* < 0.001), increased upon TS compared to CTR conditions. Finally, we found significant upregulation of *Gja1* (*p* < 0.05) and a significant downregulation of *Aqp1* (*p* < 0.05). Our data demonstrate that merely 2 weeks of reduced physical activity induce changes in genes associated with cardiac structure and electrophysiology. Hence, these data should find the basis for novel research directed to evaluate the interplay of cardiac functioning and physical inactivity.

## Graphical abstract

Hindlimb unloading does not change cardiomyocyte cross‐sectional area. Genes related to cardiac structure (e.g., Tgfb1, Srf) significantly increase upon hindlimb suspension. Genes related to increased protein turnover (e.g., Trim63) and electrophysiology (e.g., Gja1) significantly increase upon hindlimb suspension.

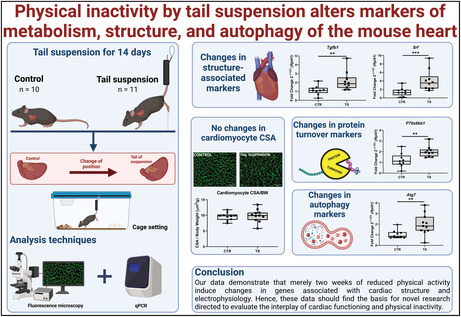

## 1 INTRODUCTION

Sedentary behavior (SB) has become a fixture in our lives as the periods of physical inactivity have increased in the last decades (Young et al., 2016). SB is defined as any waking behavior with an energy expenditure lower than 1.5 metabolic equivalents of task (Same et al., 2016; Young et al., 2016). There are many everyday activities that would comply with this definition (e.g., motorized transport, watching television, reading, using a computer, sitting at a desk) (Same et al., 2016; Young et al., 2016). It has been estimated that adults spent 6–8 h/day of SB, which would mean that even complying with the recommended 150–300 min/week of physical activity (PA), adults are still exposed to sedentary life health consequences (Lavie et al., 2019; Young et al., 2016). Reports show subpar levels of PA in the adult population, since not even 60% of the world adult population meet the minimum requirement of daily PA (Francula‐Zaninovic & Nola, 2018; Lavie et al., 2019). SB has been linked with increased morbidity and mortality independently from levels of moderate‐to‐vigorous physical activity (Same et al., 2016). Although SB has a greater prevalence in older adults, it also endangers the general population of children and adolescents (Young et al., 2016). In these ranges of age, it has been proven that less SB and higher levels of PA lead to an increased health‐related quality of life (Wu et al., 2017). Current research points to a need to reduce SB as it is linked with several diseases (e.g., type 2 diabetes, metabolic syndrome, mental health, cancer, and cardiovascular diseases [CVD]) (Lavie et al., 2019). Studies suggest that high levels of SB (i.e., prolonged sitting) are related to increased CVD incidence and mortality (De Rezende et al., 2014). While PA is a wide‐known preventive measure for CVD, it has been found that SB is associated with a greater risk for CVD independent of PA levels (Carter et al., 2017; De Rezende et al., 2014; Lavie et al., 2019). Further, SB has been connected with traditional CVD risk factors, such as body mass index, waist circumference, blood pressure, and lipid profile (De Rezende et al., 2014).

When the heart is subjected to continued increase in blood pressure or volume, the heart mass increases through an enlargement of cardiomyocyte (CM), known as cardiac hypertrophy (Bernardo et al., 2010; Guasch et al., 2013; Nakamura & Sadoshima, 2018; Oldfield et al., 2019). Cardiac hypertrophy can be distinguished into physiological versus pathological hypertrophy and show defined phenotypes, which dependent on type, duration, and magnitude of the increase workload for the heart (Oldfield et al., 2019).

Physiological hypertrophy is of an adaptive nature, which means that it is reversible and will not lead to CVD like heart failure (Samak et al., 2016). In contrast, pathological hypertrophy is characterized as heart growth associated with cardiac dysfunction (e.g., arrhythmia, myocardial infarction, heart failure) (Jalife & Kaur, 2015; McMullen & Jennings, 2007; Oldfield et al., 2019). In a final stage, pathological hypertrophy results in ventricular dilation and wall thinning accompanied by excessive deposits of extracellular matrix, also known as fibrosis (Nakamura & Sadoshima, 2018; Roemers et al., 2019; Samak et al., 2016). This fibrotic phenotype can impede the electrophysiological coupling of cardiomyocytes, thus, disturbing the propagation of the impulses and generating uneven contractions (Jalife & Kaur, 2015).

Tail suspension (TS), often used to emulate weightlessness as well as physical inactivity, has been widely used to investigate the effects of these situations on the heart. The findings reported vary among the published studies, nonetheless all point to a decompensation of cardiac structure and its electrophysiology. Studies report deficiencies in blood pressure, ejection factor, heart rhythm along with diminished cardiomyocyte cross‐sectional area (Liu et al., 2015; Martel et al., 1994; Respress et al., 2014; Zhong et al., 2016). Over the past few decades, the TS model has been studied with different timespans. The results vary in relation with the timespan chosen for each experiment, however there are a few contradicting results, for example, Martel et al. found modifications in the rats' hearts after 24 h of TS, whereas Fagette et al. did not find any changes in rats after 3 days of TS (Fagette et al., 1995; Martel et al., 1994). More recent research shows that longer timespans (i.e., 28 and 56 days) cause more severe changes in the heart that led to cardiac dysfunction (Respress et al., 2014). Given the disparities in the published data, we consider that exploring the timepoint, at which changes start to occur in the heart is key to develop potential treatments. To our knowledge, our study is the first to investigate metabolic markers in the heart muscle in combination with structural, electrophysiological, and autophagy relater markers.

SB opens the door to many ailments, especially CVD, and the relevant field that studies the effects of SB on heart adaptations is just emerging. To further investigate the effect of physical inactivity on CVD, we used an established TS mouse model that has been mainly used to induce physical inactivity phenotypes (Lavie et al., 2019; Roemers et al., 2019). Using this model, we aim to further explore how physical inactivity contributes to cardiac remodeling. To this end, we examined the expression of structural, electrophysiological, and protein turnover markers in the mouse heart, which add to present knowledge of heart modification upon SB (Liu et al., 2015; Martel et al., 1994; Respress et al., 2014; Zhong et al., 2016). Hence, our results will extend the understanding of the changes a heart undergoes when subjected to SB and its possible implications for maladaptive cardiac adaptations.

## 2 METHODS

### 2.1 Animals

Eight‐week‐old male C57BL/6J mice were housed in a conventional animal facility of the KU Leuven at 22–24°C under a 14‐h light/10 h dark cycle. Standard chow and non‐wetting water gels (HydroGel; ClearH_2_O) were supplemented ad libitum on the cage floor to permit easy access. The mice used in this experiment are the same ones used in an already published article by our group (Vanmunster et al., 2022). All mice were examined daily, and bodyweights were measured every 2 days to monitor animals' health and well‐being. Mice were anesthetized with Ketamine before sacrificing (solution 1 ml Ketamine plus 0.5 ml Xylazine in 8.5 ml saline; 100 μl of this solution per 10 g body weight i.p. administered). Sacrificing was done by cervical dislocation upon anesthesia, controlled by breathing frequency and depth, tail reflex as well as paw cutting reflex. All animal experiments and procedures such as mice feeding, maintenance, and tissue extraction were performed with the approval of the Animal Ethics Committee of the KU Leuven, Leuven, Belgium (P110/2018) and done in accordance with the “Directive 2010/63/EU” of the European Parliament.

### 2.2 Interventions

Mice were randomly divided into two groups: (1) sedentary control (CTR, *n* = 10), which remained untouched for the duration of the experiment; (2) tail suspension (TS; *n* = 11), mice of this group were subjected to tail suspension for 14consecutive days. In brief, by means of strong duct tape (tesa®) and medical adhesive tape a ring was carefully attached to the tail of the mice. The cages were covered by Plexiglas plates with on which we had fixed curtain rods. The ring was threaded with the curtain roads, thereby preventing the hindlimbs from touching the bottom of the cage. The bottom of each cage was lined with cork plates (8 mm thick) providing a robust, but comfortable surface with texture ideal for easy grip. This combination of both the curtain rods and the cork plates allowed the mice to move freely across the full length of the cage while reducing the external mechanical loading on the hindlimbs. We sacrificed the mice at the same time point after 14 days of TS and then the hearts were excised. The hearts were dissected in two in the transversal plane. Half of the tissue was snap‐frozen in liquid N for biochemical analysis and the other half was embedded in freezing media for histological analysis. This dissection method allowed us to study the ventricles in both histology (upper half) and biochemical analysis (lower half).

### 2.3 Immunohistochemistry

Immunohistochemical analysis was adapted from a previously described protocol (Greiwe et al., 2015). Briefly, 7‐μm‐thick cryosections were warmed to room temperature (RT) and subsequently fixed for 10 min in −20°C pre‐cooled acetone. After drying for 20 min the slides were incubated for 60 min at 37°C with an antibody for Laminin (dilution: 1:500 in PBS 0.5% bovine serum albumin (BSA), Sigma, L9393). Afterward, slides were rinsed three times for 5 min each with PBS and then incubated for 60 min at 37°C with the appropriate polyclonal secondary antibody (Donkey Anti‐Rabbit IgG H&L Alexa Fluor 488, dilution: 1:500 in PBS 0.5% BSA; Abcam, ab150073). The sections were then washed three times 5 min each in PBS. The slides were incubated for 15 min at RT with Hoechst (dilution 1:5000 in PBS 0.5% BSA, Sigma, 33,342). The sections were washed three times for 5 min each and mounted with Dako Fluorescence Mounting Medium (Dako, S3023) and supplied a coverslip. All slides were examined with a Nikon Eclipse E1000 microscope and compatible NIS Elements software (40× magnification). Fiji was the software used for data acquisition. Eighty CM of each heart was manually measured (Schindelin et al., 2012).

### 2.4 Picrosirius red staining

Briefly, 7‐μm‐thick cryosections of heart tissue were warmed to RT and fixed in 70% ethanol for 3 min. The samples were hydrated in dH_2_O for 5 min and stained in 0.1% Picrosirius Red (Abcam, ab246832) for 1 h. The slides were washed twice for 5 min in 0.5% acetic acid. Samples were subsequently dehydrated in 100% ethanol for 5 min and mounted with DPX (Merk, 06522) and a coverslip. All pictures were acquired with Olympus IX83 microscope at magnification 40×.

Fibrosis area (μm^2^) was quantified using MRI fibrosis tool and Color Deconvolution plugin for ImageJ (https://github.com/MontpellierRessourcesImagerie/imagej_macros_and_scripts/wiki/MRI_Fibrosis_Tool). Four photos were analyzed per animal. The data obtained were then averaged to use in statistical tests.

### 2.5 RNA extraction and reverse transcription

Total RNA was extracted from 15 to 20 mg of heart using TRI Reagent® (Molecular Research Center) as per manufacturer's protocols. Quantity and quality of RNA were assessed by a spectrophotometer (SimpliNano, Biochrom). One microgram of total RNA was then used for reverse transcription (RT) with a QuantiTect Reverse Transcription Kit (Qiagen). RNA integrity was controlled by RNA gel electrophoresis as described (Dalle et al., 2021; Mathes et al., 2019).

### 2.6 Real‐time qPCR analysis

Quantitative real‐time reverse transcriptase PCR was performed by mixing the following components: (1) 12.5 μl GoTaq(R) qPCR Master Mix (Promega); (2) 0.25 μl of CXR Reference Dye (Promega); (3) 100 nM primer mix consisting of primers (sense and antisense: see Table 1); (4) 8.25 μl nuclease‐free H_2_O to reach a final reaction volume of 25 μl (Stein & Wade, 2005). The reaction was performed on a QuantStudio 3 Real‐Time PCR System (Thermo Fisher Scientific). The quantity of the gene of interest in each sample was normalized to that of *Rpl41* using the comparative (2‐∆∆CT) method (Mathes et al., 2019; Perez et al., 2017; Pilbrow et al., 2008). Unbiased amplicon generation by qPCR reactions was finally controlled by DNA gel electrophoresis as described (Dalle et al., 2021; Mathes et al., 2019).

**TABLE 1:** Primer sequences

Gene symbol	Accession number	Sense primer	Antisense primer	Amplicon size [bp]
*Acta1*	NM_009606.3	CAGAGTCAGAGCAGCAGAAAC	AGCCGTTGTCACACACAAGA	75
*Aqp1*	NM_007472.2	ACATTGTGAACCGAGAGCCA	GTGTCTGCTAGGGAACGGAG	116
*Aqp7*	NM_007473.4	CTGCCCTACTGACCTCTCCC	GCTGGTCTTATGAAGTAGGTTCTC	220
*Atg7*	NM_001253717.1	CTGGAAAATTCCCACGAGCACA	GCTACTGTTCTTACCAGCCTCAC	114
*Atp2a2*	NM_009722.3	GGAGAATATCTGGCTCGTGGG	GTTACTCCAGTATTGCGGGTTG	218
*Bcl2*	NM_009741.5	CATCACTCTGGGTGCATACCT	GGAGTTTCGGTGGAACTGTCTT	316
*Becn1*	ENSMUST00000130916.8	ATGGAGGGGTCTAAGGCGTC	CCGGTCCAGGATCTTGAAGC	105
*Col1a1*	NM_007742.4	GGTTCGTGACCGTGACCTTG	CGATCCAGTACTCTCCGCTCT	161
*Gja1*	NM_010288.3	ACAGGTCTGAGAGCCCGAAC	TGTCTGGGCACCTCTCTTTCAC	112
*Fbxo32*	NM_026346.3	TCAGAGAGGCAGATTCGCAAG	TGAGGGGAAAGTGAGACGGA	218
*Fn1*	NM_010233.2	ATCACAGTAGTTGCGGCAGGAG	TGGGAGGAGGGACAGCCGTTT	187
*Map1lc3b*	NM_026160.5	ACCAAGATCCCAGTGATTATAGAG	GTCTCCTGCGAGGCATAAA	269
*Mtor*	NM_020009.2	TGCGGCAGGATGAACGAGTG	GCCCGAGTTGGTGGACAGAG	131
*Nppa*	NM_008725.3	GACCACCTGGAGGAGAAGATG	AGAGGGCAGATCTATCGGAGG	199
*Nppb*	NM_008726.6	TGGGCTGTAACGCACTGAA	GACCCAGGCAGAGTCAGAAA	78
*P70s6kb1*	NM_001114334.2	CACGAACACCTGTCAGCCCA	CCCCGCTCACTGTCACATCC	147
*Slc8a1*	NM_011406.3	CTATTGAAGGCACAGCCCGA	GCCACCAAGCTCATTCAACA	215
*Srf*	NM_020493.2	CAGTGGGGAAACCAAGGACACA	GTGCTGTCTGGATTGTGGAGG	83
*Tgfb1*	NM_011577.2	AACAATTCCTGGCGTTACCTTG	CCCTGTATTCCGTCTCCTTG	122
*Tnfa*	NM_013693.3	GCCCACGTCGTAGCAAACC	TGAAGAGAACCTGGGAGTAGACA	154
*Trim63*	NM_001039048.2	GGTGCCTACTTGCTCCTTGT	ACCTGGTGGCTATTCTCCTTG	201
*Ulk1*	ENSMUST00000200299.2	TGAGGTCATTATGTCCCAGCAC	GTTCTTCTCATAAAACAGGCGCAA	139
*Yap1*	NM_001171147.1	TCGGCAGGCAATACGGAATATCAA	GCATTCGGAGTCCCTCCATC	114

### 2.7 Western blotting

According to established protocols, ~5 mg of frozen heart tissue was homogenized 5 × 20 s at 6.0 m/s in ice‐cold lysis buffer (Cell Signaling) using a FastPrep‐24™ Classic Instrument (MP Biomedicals) (Dalle et al., 2021). Homogenates were then centrifuged at 10,000*g* for 25 min at 4°C and the supernatant was collected and immediately stored at −80°C. The homogenates' protein contents were determined by using the DC protein assay kit (Bio‐Rad laboratories). Fifteen of homogenate protein was separated by SDS‐PAGE (8% gels) and transferred to a polyvinylidene difluoride (PVDF) membrane. The membranes were blocked either in 5% bovine serum albumin (BSA) in TBS‐T or in 5% low‐fat milk in TBS‐T (depending on the one used to dilute the antibody) for 1 h and afterward incubated overnight at 4°C with the following antibodies: mTOR (1:1000, #2972, Cell Signaling); phospho‐mTOR (Ser2448) (1:1000, #2971, Cell Signaling); P70S6K1 (1:1000, #9202, Cell Signaling); phospho‐P70S6K1 (Thr421/Ser424) (1:1000, #9204 S, Cell Signaling) dissolved in 5% BSA in TBS and Vcl (1:2000, V9131, Sigma) dissolved in 5% low‐fat milk in TBS‐T. Afterward, the membranes were incubated for 1 h at RT with horseradish peroxidase‐conjugated anti‐mouse or anti‐rabbit secondary antibodies (1:10,000 both in 5% low‐fat milk in TBS‐T for both, Sigma) for chemiluminescent detection. Membranes were scanned and quantified with Genetools and Genesnap software (Syngene, Cambridge), respectively.

### 2.8 Statistics

All statistics were performed using Prism 8 (GraphPad Inc.,). Values for CTR and TS are plotted in box plots. Normality was assessed using the D'Agostino & Pearson (*p* < 0.05). Depending on the outcome of the normality test, differences between CTR and TS were analyzed using either the unpaired Student's *t* test or Mann–Whitney U test. Levels of significance were *p < 0.05, **p < 0.01, and ***p < 0.001. Some gene expression presented statistical outliers that were identified using ROUT method (Q = 1%). In Figure S1 includes scatter graphs that include the outliers marked in red for CTR and blue for TS. To evaluate if outliers modify significance, they were removed, and statistical analysis was performed on clean data. The results of this are reflected in Table S1.

## 3 RESULTS

### 3.1 Cardiomyocytes cross‐sectional area does not change upon tail suspension

**FIGURE 1:**
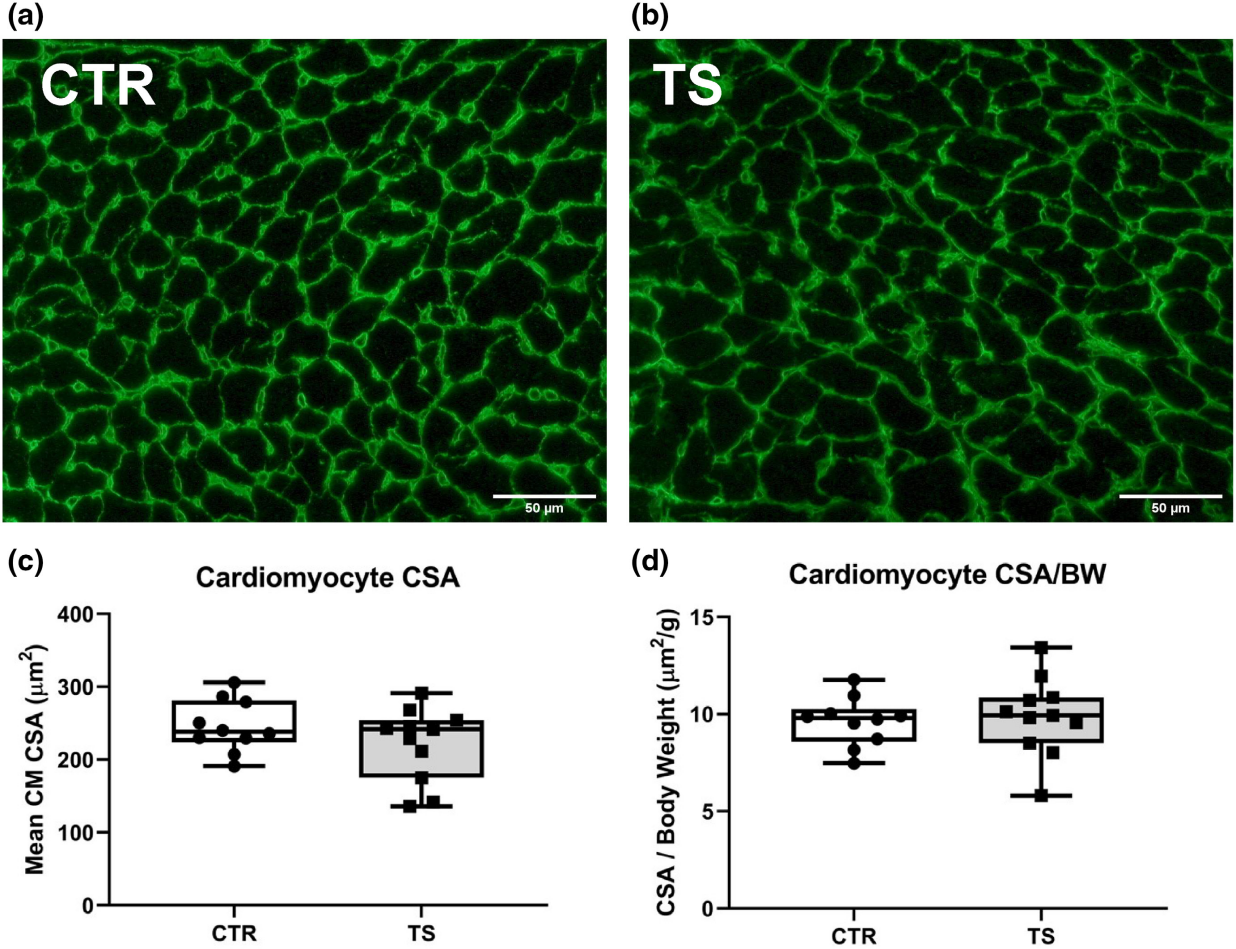
Cross‐sectional area assessment of heart cardiomyocytes upon tail suspension. (a) Histological section of the heart from a control (CTR) condition. (b) Histological section of the heart from a tail suspension (TS) condition. (c) Assessment of the mean cardiomyocyte (CM) cross‐sectional area (CSA) between control (CTR) and tail suspension (TS) conditions. (d) Assessment of the mean cardiomyocyte cross‐sectional area related to body weight (BW) between control (CTR) and tail suspension (TS) conditions. Bar = 50 μm. 40× magnification. Eighty CM was counted by animal. All experiments were performed with CTR = 10 mice; TS = 11 mice. Normality was assessed with D'Agostino and Pearson and depending on the outcome, differences between CTR and TS were analyzed using either the unpaired Student's *t* test or Mann–Whitney U test.

Measurement of cardiomyocytes cross‐sectional areas (CSA) is a widely used method to assess heart overall condition and specifically to study hypertrophy or atrophy (Shimizu & Minamino, 2016). Cardiomyocyte CSAs were evaluated after 14 days of tail suspension (Figure 1a,b). There are no apparent changes in CSA between control and tail suspension (Figure 1c) nor in CSA indexed with bodyweight (Figure 1d). Bodyweights can be found in Table S2.

### 3.2 Tail suspension does not induce pathological hypertrophy

**FIGURE 2:**
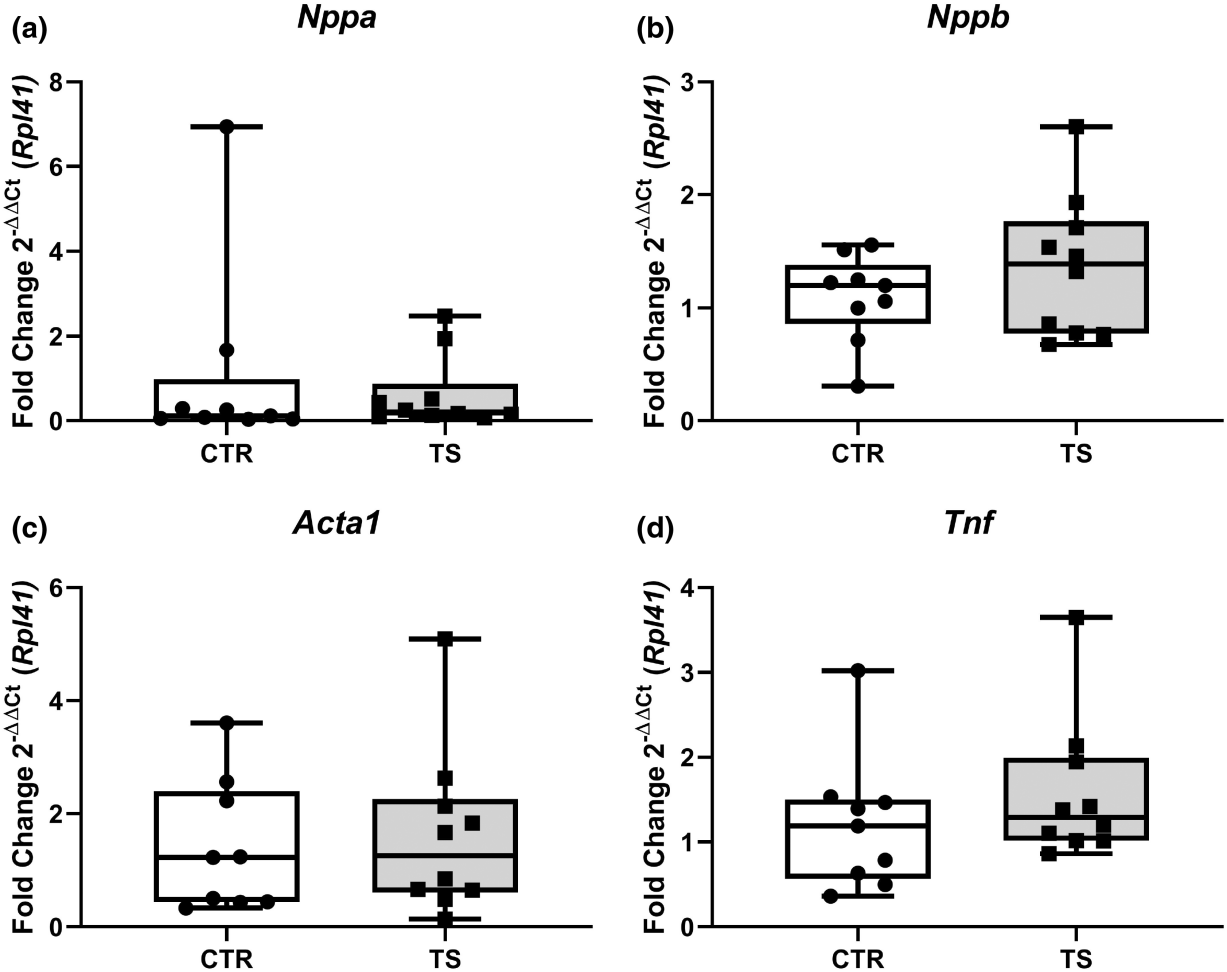
Analysis of cardiac damage and pathological hypertrophy genes. (a) *Atrial natriuretic peptide* (*Nppa*); (b) *Brain natriuretic peptide* (*Nppb*); (c) *α‐skeletal muscle Actin* (*Acta1*); (d) *Tumor necrotic factor α* (*Tnf*). CTR = control condition; TS = tail suspension condition. All experiments were performed with CTR = 9 mice; TS = 10 mice. Normality was assessed with D'Agostino and Pearson and depending on the outcome, differences between CTR and TS were analyzed using either the unpaired Student's *t* test or Mann–Whitney U test.

Long periods of tail suspension in mice (i.e., 28–56 days) have been associated with cardiac damage (Liang et al., 2019; Liu et al., 2015). Markers like *natriuretic peptide A* (*Nppa*, Figure 2a), *natriuretic peptide B* (*Nppb*, Figure 2b), *actin alpha 1* (*Acta1*, Figure 2c) or *tumor necrotic factor α* (*Tnf*, Figure 2d) are classically studied to assess cardiac damage and pathological hypertrophy (Djalinac et al., 2020; Jalife & Kaur, 2015; Wang et al., 2020). As represented in Figure 2, we did not observe any changes in these genes associated with pathological hypertrophy.

### 3.3 Tail suspension does not cause an upregulation on fibrotic markers

**FIGURE 3:**
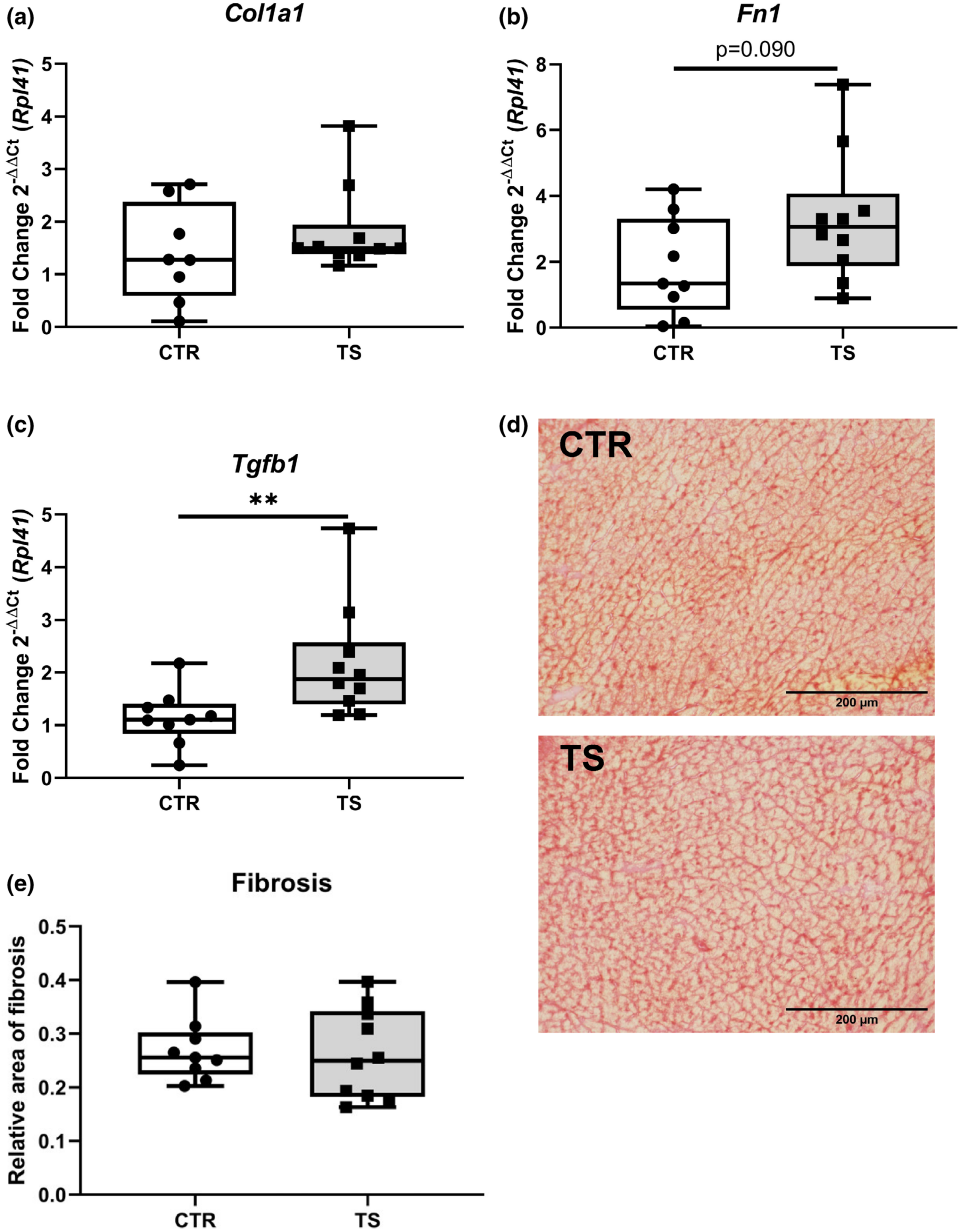
Analysis of fibrosis‐related genes in cardiomyocytes. (a) *Collagen 1* (*Col1a1*); (b) *Fibronectin* (*Fn1*); (c) *Transforming growth factor beta 1* (*Tgfb1*). CTR, control condition; TS, tail suspension condition. (d) Picrosirius Red staining of CTR and TS conditions. (e) Quantification of fibrosis area (μm^2^). Bar = 200 μm. 40× magnification. All experiments were performed with CTR = 9 mice; TS = 10 mice. Normality was assessed with D'Agostino and Pearson and depending on the outcome, differences between CTR and TS were analyzed using either the unpaired Student's *t* test or Mann–Whitney U test. Levels of significance were **p < 0.01.

Fibrosis constitutes one of the most common irreparable damages that occurs in the heart in the event of a CVD. Therefore, we analyzed classical fibrosis‐related genes, that is, *collagen 1* (*Col1a1*), *fibronectin* (*Fn1*), and *transforming growth factor beta 1* (*Tgfb1*) (Jalife & Kaur, 2015; Travers et al., 2016). *Col1a1* expressions did not change (Figure 3a). In contrast, *Fn1* expressions showed a clear tendency of upregulations (*p* = 0.090, Figure 3b) and *Tgfb1* demonstrated significantly (*p* < 0.01) upregulation in expression profiles upon TS compared to CTR (Figure 3c). To get a better understanding of the increase in *Tgfb1* in TS mice a Picrosirius Red staining of the hearts was performed (Figure 3d,e). Upon quantification of fibrosis, there were no significant differences between TS and control mice.

### 3.4 Tail suspension increases expression of cell regeneration markers

**FIGURE 4:**
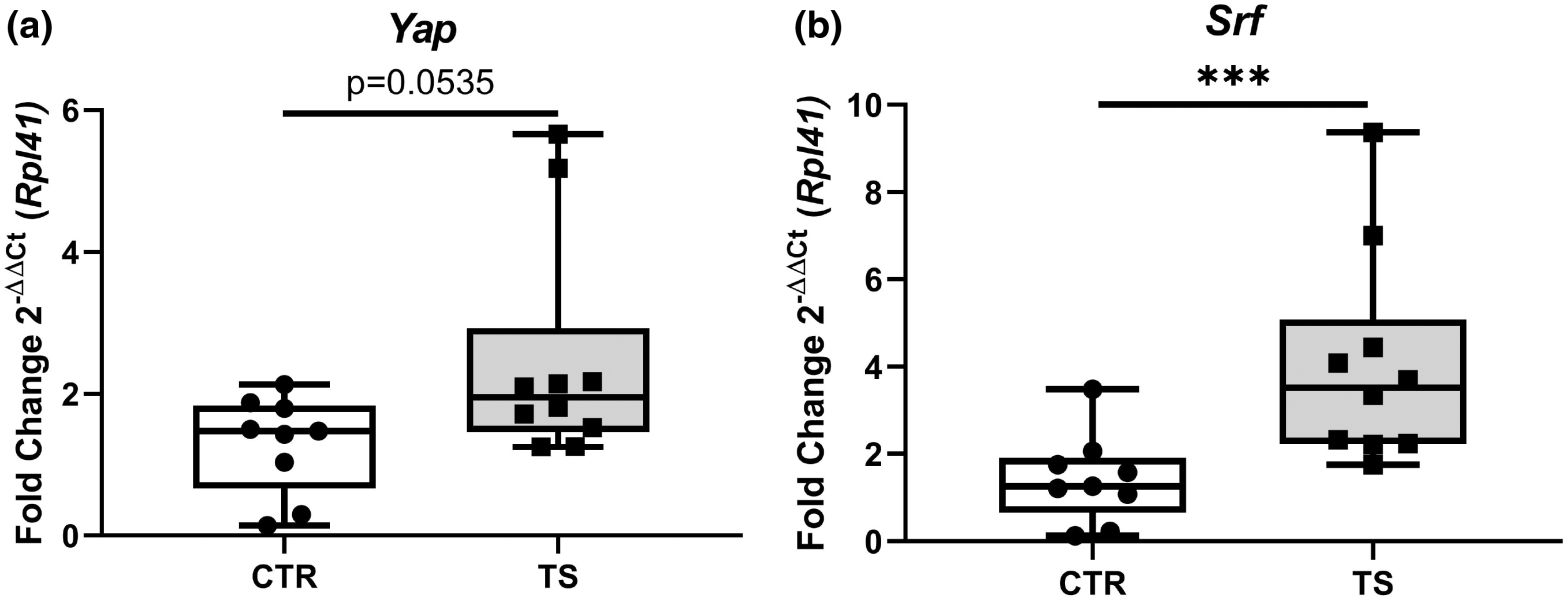
Assessment of cardiac cell regeneration markers. (a) *Yes‐associated protein* (*Yap*); (b) *Serum response factor* (*Srf*). CTR, control condition; TS, tail suspension condition. All experiments were performed with CTR = 9 mice; TS = 10 mice. Normality was assessed with D'Agostino and Pearson and depending on the outcome, differences between CTR and TS were analyzed using either the unpaired Student's *t* test or Mann–Whitney U test. Levels of significance were ***p < 0.001.

Cell survival and cardiomyocyte growth are both crucial for physiological hypertrophy. (Nakamura & Sadoshima, 2018) The expressions of genes such as *Yes‐associated protein* (*Yap*, Figure 4a) and *serum response factor* (*Srf*, Figure 4b), of which the latter was upregulated significantly (*p* < 0.001) on tail‐suspended mice (Camberos et al., 2019; Davis et al., 2002; Nakamura & Sadoshima, 2018; Wang et al., 2018). *Yap* showed a pronounced tendency for upregulation, even if not statistically significant (*p* = 0.0535) YAP is a known component of the Hippo pathway that also has been known to interact with SRF (Wang et al., 2018).

### 3.5 Tail suspension changes protein turnover balance

**FIGURE 5:**
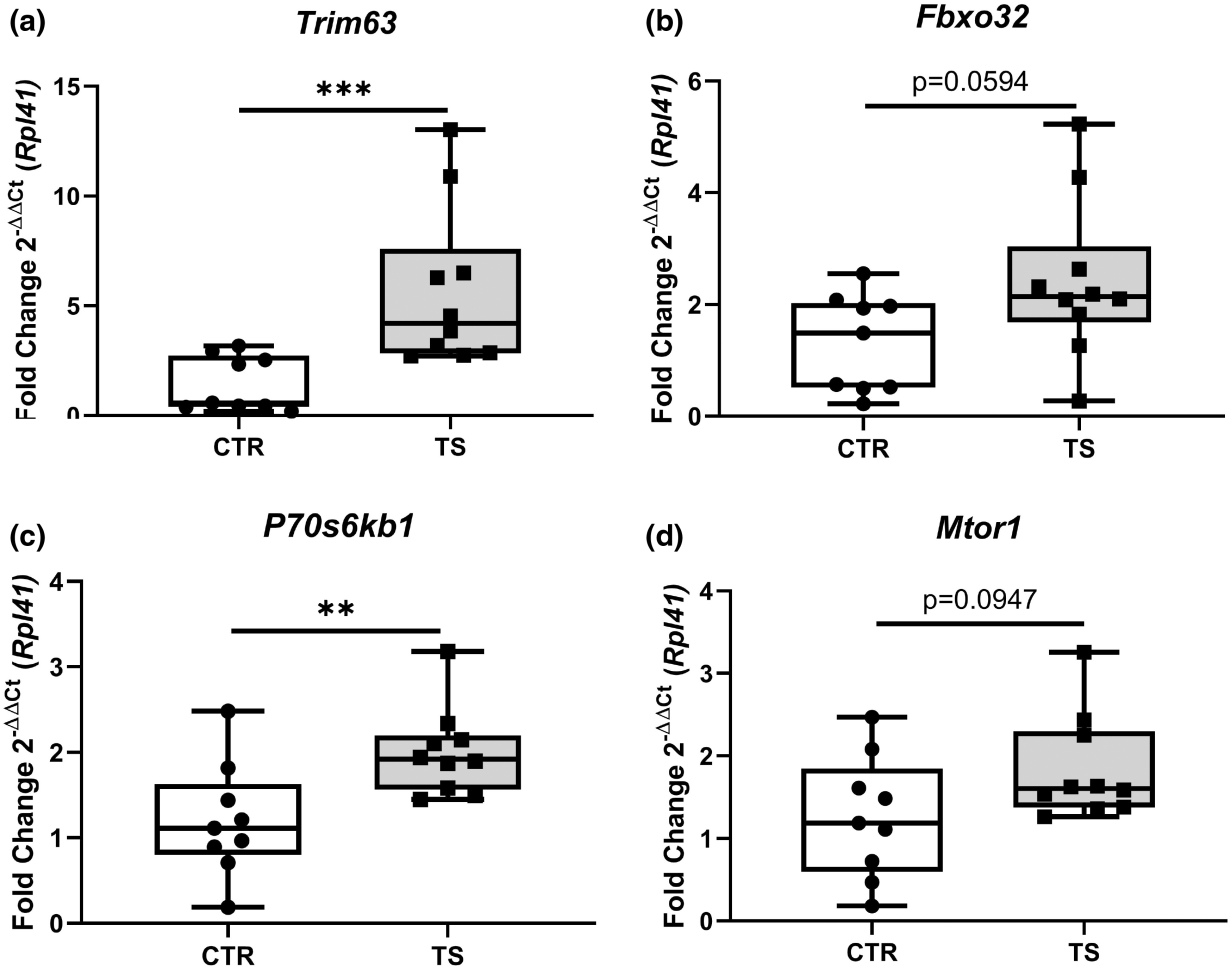
Investigation of protein degradation‐ and protein synthesis‐related genes. (a) *Tripartite Motif Containing 63* (*Trim63*); (b) *F‐Box Protein 32* (*Fbxo32*); (c) *Ribosomal protein S6 kinase* (*P70s6kb1*); (d*) Mechanistic target of rapamycin* (*Mtor1*). CTR, control condition; TS, tail suspension condition. All experiments were performed with CTR = 9 mice; TS = 10 mice. Normality was assessed with D'Agostino and Pearson and depending on the outcome, differences between CTR and TS were analyzed using either the unpaired Student's *t* test or Mann–Whitney U test. Levels of significance were **p < 0.01, and ***p < 0.001.

**FIGURE 6:**
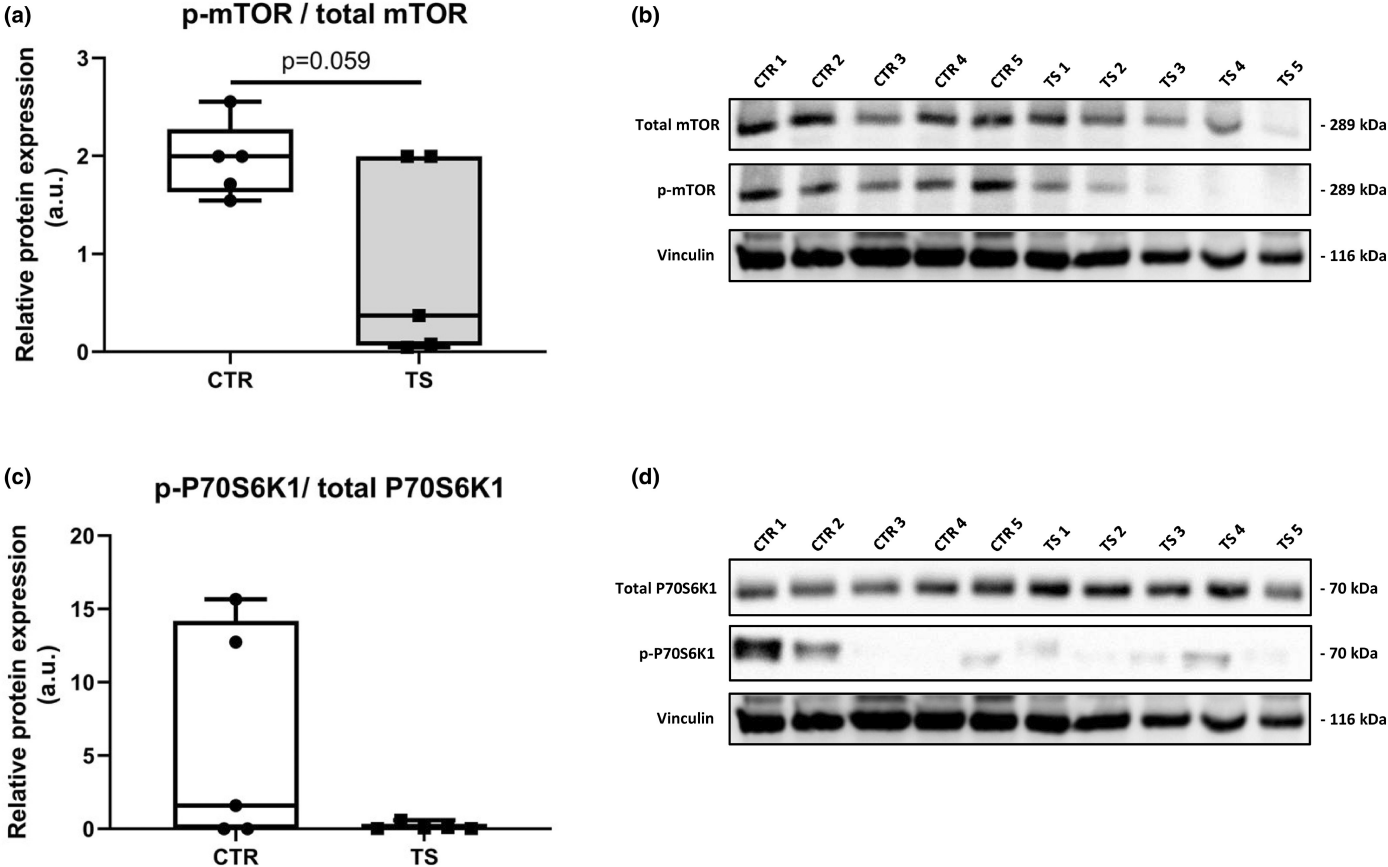
Evaluation of protein synthesis. (a) Phospho‐mTOR (Ser2448)/ total mTOR; (b) Bands quantified of p‐mTOR/ total mTOR normalized with Vinculin (1) (corresponds to loading control for total proteins) and Vinculin (2) (corresponds to loading control for p‐mTOR); (c) Phospho‐P70S6K1 (Thr421/Ser424)/ total P70S6K1; (d) Bands quantified of p‐P70S6K1/total P70S6K1 normalized with Vinculin (3) (corresponds to loading control for phosphorylated proteins). CTR, control condition; TS, tail suspension condition. All experiments were performed with CTR = 5 mice; TS = 5 mice. All gels were done simultaneously. Normality was assessed with D'Agostino and Pearson and depending on the outcome, differences between CTR and TS were analyzed using either the unpaired Student's *t* test or Mann–Whitney U test. Level of significance is *p* < 0.05.

It has been demonstrated that the heart suffers from atrophy when subjected to long‐term tail suspension (Respress et al., 2014; Zhong et al., 2016, 2021). To investigate whether our short‐term tail suspension exerts atrophy‐related effects at the molecular level, we tested the atrophy‐related genes *Trim63* and *Fbxo32* (Bernardo et al., 2018). *Trim63* (Figure 5a) was upregulated (*p* < 0.001) and *Fbxo32* (Figure 5b) had a clear tendency of increased expression (*p* = 0.0594). We further tested genes involved in protein synthesis through the analysis of the genes *P70s6kb1* as well as *Mtor1 (*Nakamura & Sadoshima, 2018). We found increased *P70s6kb1* expressions (*p* < 0.01, Figure 5c), whereas *Mtor1* did not change (Figure 5d) upon TS compared to CTR condition. To evaluate the activation of the protein synthesis pathway, p‐mTOR (Ser2448)/total mTOR and p‐P70S6K1 (Thr421/Ser424)/total P70S6K1 ratios were examined. There were no differences in total mTOR and total P70S6K1 between TS and CTR (data not shown). There is, however, a clear decrease in p‐mTOR/ total mTOR in TS mice (Figure 6a,b), although the difference is not significant. There was no difference in p‐P70S6K1/ total P70S6K1 (Figure 6c,d) between TS and CTR mice.

### 3.6 Tail suspension promotes autophagy‐related markers

**FIGURE 7:**
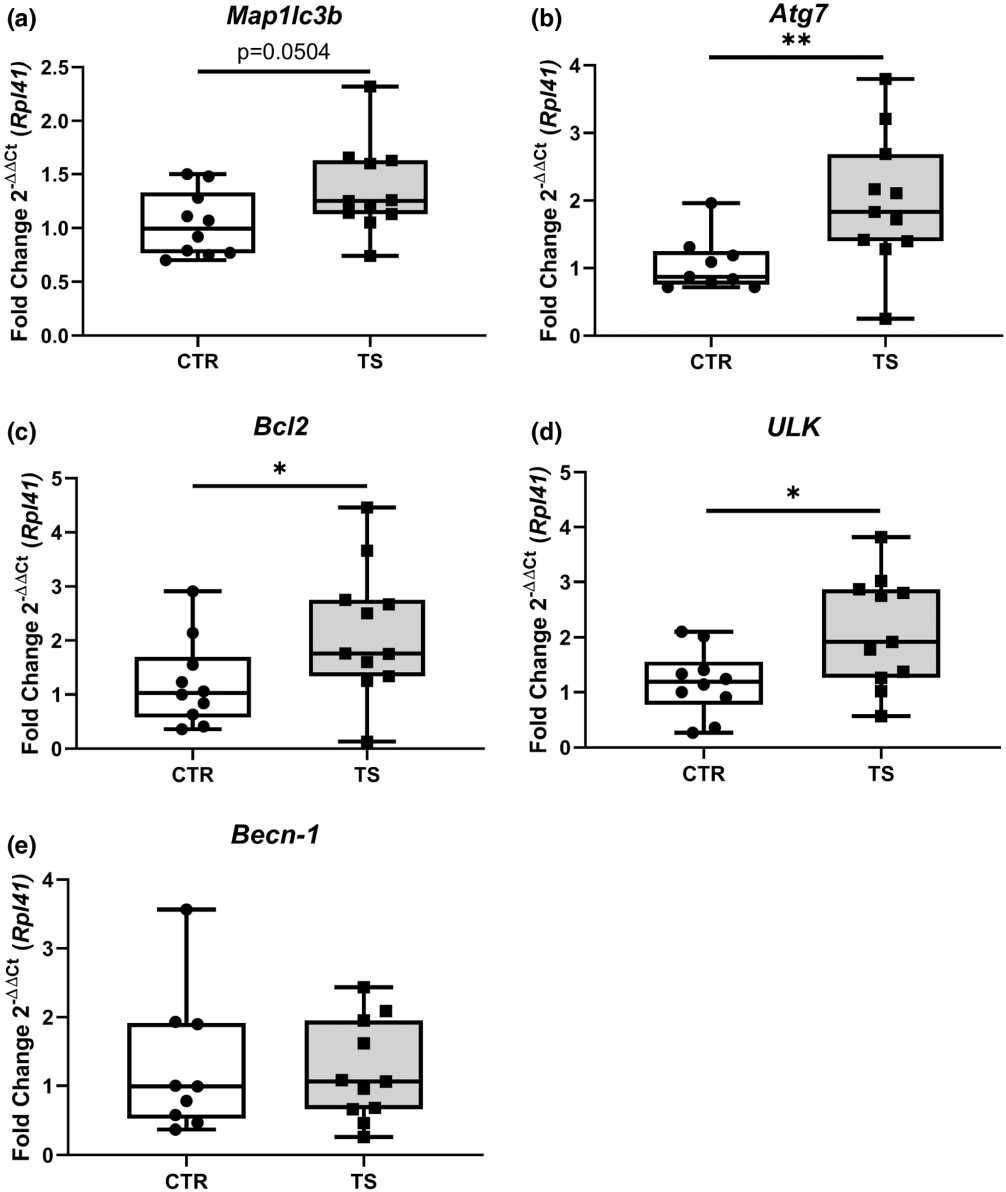
Evaluation of autophagy genes expression. (a) *Microtubule Associated Protein 1 Light Chain 3 Beta* (*Map1lc3b*); (b) *Autophagy Related 7* (*Atg7*); (c) *BCL2 Apoptosis Regulator* (*Bcl2*); (d) *Unc‐51 Like Autophagy Activating Kinase 1* (*ULK*); (e) *Beclin‐1* (*Becn‐1*). CTR, control condition; TS, tail suspension condition. All experiments were performed with CTR = 10 mice; TS = 11 mice. Normality was assessed with D'Agostino and Pearson and depending on the outcome, differences between CTR and TS were analyzed using either the unpaired Student's *t* test or Mann–Whitney U test. Levels of significance were *p < 0.05, **p < 0.01.

A previous study by Liu et al. (2015) demonstrated that autophagy was increased in TS, thus producing cardiac muscle wasting (Liu et al., 2015). The expression of five autophagy markers (i.e., microtubule association protein one light chain 3β (Map1lc3b), UNC‐51 like autophagy activating kinase 1 (ULK1), autophagy‐related 7 (Atg7), BCL2 apoptosis regulator (Bcl2) and Beclin‐1 (Becn‐1)) was assessed (Dikic & Elazar, 2018). There is trend of increased expression of *Map1lc3b* (Figure 7a) even if not significant. There is a significant increase in *Atg7* (Figure 7b), *Bcl2* (Figure 7c), and *ULK* (Figure 7d) *Becn‐1* (Figure 7e) did not show any difference. These results point to a possible increase in autophagy in TS mice.

### 3.7 Tail suspension modifies connexin 43 and aquaporin 1 expression in the heart

**FIGURE 8:**
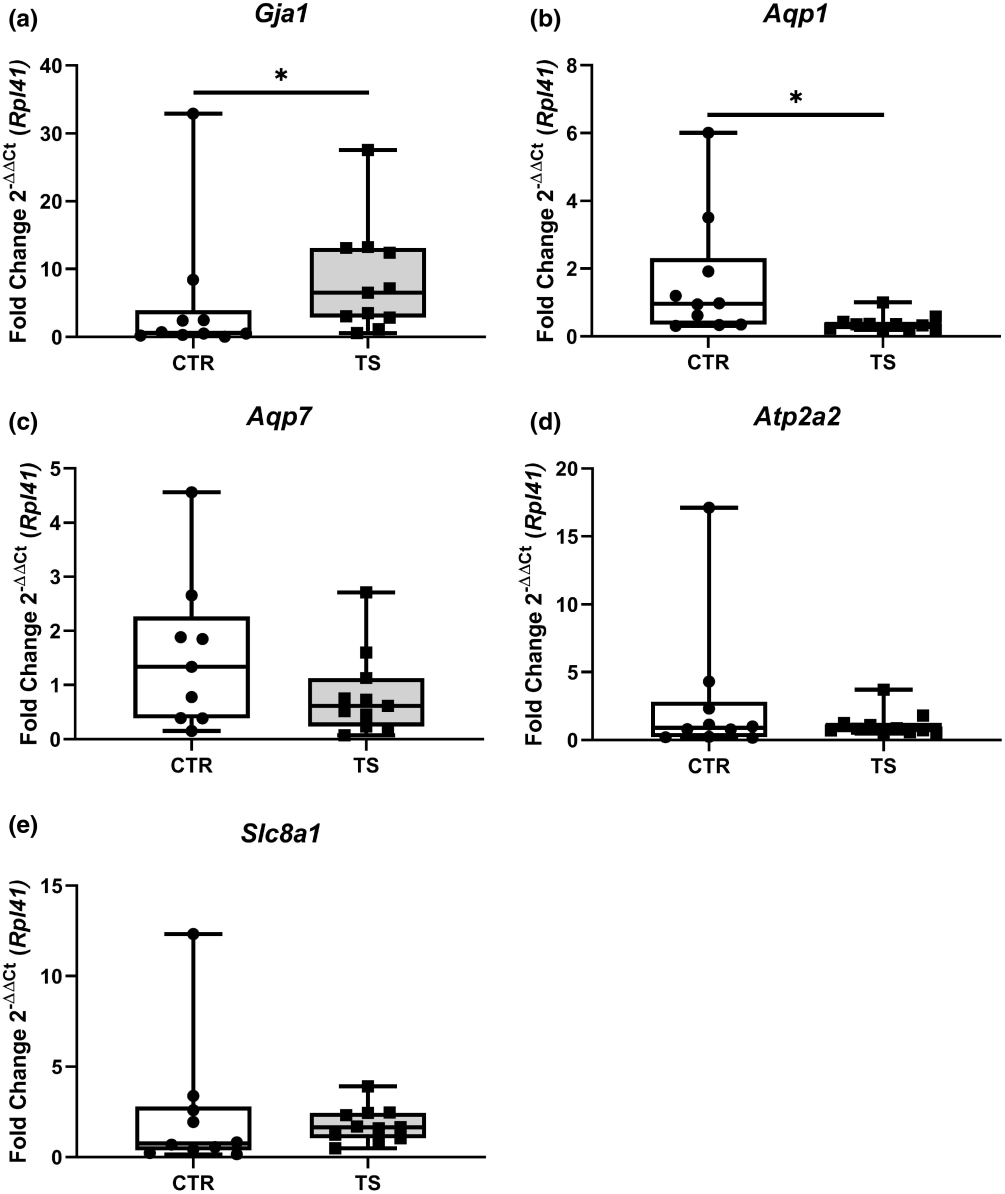
Analysis of genes involved in cardiac electrophysiological processes. (a) *Gap Junction Protein Alpha 1/connexin 43* (*Gja1*); (b) *Aquaporin 1* (*Aqp1*); (c) *Aquaporin 7* (*Aqp7*); (d) *ATPase Sarcoplasmic/Endoplasmic Reticulum Ca* ^*2+*^ *Transporting 2* (*Atp2a2*); (e) *Solute carrier family 8 sodium/calcium exchanger* (*Slc8a1*). CTR, control condition; TS, tail suspension condition. All experiments were performed with CTR = 10 mice; TS = 11 mice. Normality was assessed with D'Agostino and Pearson and depending on the outcome, differences between CTR and TS were analyzed using either the unpaired Student's *t* test or Mann–Whitney U test. Levels of significance were *p < 0.05.

Cardiac structural remodeling is inevitably accompanied by electrophysiological changes. To evaluate these changes, we studied several Ca^2+^‐handling genes, that is, *gap junction protein 1* (*Gja1* also known as connexin 43, Figure 8a), *aquaporin 1* (*Aqp1*, Figure 8b), *aquaporin 7* (*Aqp7*, Figure 8c), *ATPase sarcoplasmic/endoplasmic reticulum Ca* ^*2+*^ *transporting 2* (*Atp2a2*, Figure 8d), *solute carrier family 8 member A1* (*Slc8a1*, Figure 8e) to get an insight into the electrophysiology‐managing genes of the heart (Butler et al., 2006; Nakamura & Sadoshima, 2018; Ottolia et al., 2021; Severs, 2001; Verkerk et al., 2019). The expression of *Gja1* was significantly upregulated (*p* < 0.05) while *Aqp1* was significantly downregulated (*p* < 0.05) in TS mice. *Slc8a1*, *Aqp7*, and *Atp2a2* remained unchanged.

## 4 DISCUSSION

The heart is a dynamic muscle, which main objective is to maintain perfusion to the peripheral organs, thus, it needs to be capable of adapting to myriad of stimuli. Here, we demonstrate that 2 weeks of tail suspension results in changes in genes regulating various parts of crucial cardiac subsystems, such as cardiac fibrosis and damage, protein turnover, and cell survival as well as electrophysiology.

When the heart is subjected to TS, an intervention used to recreate SB by imitating orthostatic positions in humans, the heart will have to undergo changes to compensate for the different position (Lavie et al., 2019; Roemers et al., 2019). This model is also commonly used to generate a microgravity in the chest cavity to simulate space flight. Both situations put the heart in strain. Lack of PA associated with these conditions (i.e. space flight or SB) has been associated with loss of heart mass and atrophy of the cardiac muscle (Martel et al., 1998). The results reported from this model vary. Martel et al. recorded signs of transient tachycardia in rats after 24 h of TS, while Fagette et al. did not find changes in blood pressure or heart rhythm in two separate occasions (rats subjected to 3 days of TS and rats subjected to 13 days of TS) (Fagette et al., 1994, 1995; Martel et al., 1994). Nonetheless, two later studies showed impaired contractility in isolated CM from rats in TS for 7 and 14 days (Dunlap et al., 1996; Pogodina et al., 2004). Respress et al. reported an increase in susceptibility of arrhythmia due to changes in Ca^2+^ handling after 28 and 56 days of TS in mice (Respress et al., 2014). Further, two studies reported explicit signs of cardiac dysfunction and cardiac atrophy. Liu et al. described that after 8 weeks of TS in rats provoked cardiac atrophy represented by diminished CMs' CSA and a significant increase in autophagy markers. The authors speculated that these phenotypes caused the observed cardiac dysfunction (Liu et al., 2015). Liang et al. found decreased CMs' CSA and reduced heart weight in mice after 28 days of TS, which induced myocardial dysfunction (Liang et al., 2020). In our study, TS mice did not show signs of atrophy as CSA was not different from the control group. However, this discrepancy with the mentioned studies may base on the differently applied periods of TS and most dramatic changes in the heart seem to occur after 4 weeks of TS. Nevertheless, and importantly, our results demonstrate that a short period of inactivity results in moderate cardiac changes at the gene expression level.

Cardiac stress and injury are associated with upregulation of fetal genes (*Nppa, Nppb*, and *Acta1*) (Bernardo et al., 2010). However, these genes along with *Tnf* do not reveal any structural impairments upon 2 weeks of TS. It is noteworthy that at this time point, the mice did not exhibit signs of cardiac damage considering that previous literature clearly states that 14 days is enough to reduce contractility of CM (Liang et al., 2020). This could imply that fetal gene regulation and reduced CM contractility could be independent from each other upon a relatively short period of SB.

Upregulated protein synthesis is associated with cardiac hypertrophy (Nakamura & Sadoshima, 2018). In our model, we studied marker genes of protein synthesis, such as *Mtor1* and *P70s6kb1*, and we found that TS resulted in a trend of elevated *Mtor1* expression and a significant upregulation of *P70s6kb1*. Although there is no indication of either physiological or pathological hypertrophy, we identified an increase in protein synthesis‐related genes. Furthermore, we found upregulated *Trim63* and *Fbxo32* expression. These genes are associated with protein degradation and, hence, atrophy. To get a better understanding of the protein synthesis pathway we analyzed protein content and found that the activation of mTOR is markedly less in TS mice than CTR. There is no significant difference in P70S6K1, a downstream protein in the mTOR protein synthesis pathway. Cautiously, we can determine that there is a change after 14 days of TS mice at the molecular level of protein turnover, if not yet seen at the tissue level Strengthening these results, we found a significant increase in expression of several autophagy genes, specifically *Atg7*, *ULK*, and *Bcl2* which, as found in a previous study, lead to increased protein degradation and to cardiac muscle wasting (Liu et al., 2015).

Cardiac fibrosis is a relevant hallmark of pathological hypertrophy and the gene Tgfb1 is one of its preeminent markers (Travers et al., 2016). We found *Tgfb1* to be significantly upregulated in TS mice suggesting that TS generates a pathological stimulus to the heart. Interestingly and considering that TGF‐β is directly responsible for their promotion (Travers et al., 2016), *Fn1* and *Col1a1* (Jalife & Kaur, 2015) remained unchanged in the treated group (Jalife & Kaur, 2015; Travers et al., 2016). There was no fibrosis found in the TS mice hearts. These data show that 14 days of TS are not sufficient to cause fibrosis, but sufficient to modify *Tgfb1* expression. TGF‐β has been associated with the transition of adaptive to pathological hypertrophy and with the upregulation of natriuretic peptide B (Oldfield et al., 2019). Furthermore, TGF‐β‐associated pathways interacting YAP, a downstream effector of the Hippo pathway and involved in cardiac regeneration and proliferation (Travers et al., 2016), tended to be upregulated in TS mice. Interestingly, YAP has been proven to improve cardiac function and contractility and Camberos et al. found YAP to be induced in adult cardiac progenitor cells by simulated microgravity (Camberos et al., 2019; Gyöngyösi et al., 2017; Nakamura & Sadoshima, 2018; Nattel, 2017). This finding in combination with ours could indicate that cardiac unloading stimulates defined mechanisms to maintain cardiac functionality under such conditions. YAP/TAZ pathway interacts with the MRTF/SRF pathway in actin polymerization and cell proliferation in response to mechanical cues (Miano, 2010). SRF is a known actin and sarcomerogenesis manager and leads to poor contractility and mishandling of Ca^2+^, if downregulated (Miano, 2010; Niu et al., 2008). We found upregulated *Srf* expressions in TS mice, which has been detected in exercise‐induced physiological hypertrophy (Bernardo et al., 2018; Frey & Olson, 2003; Wu et al., 2019). This finding could further indicate that cardiac adaptations under SB conditions include stimulations of contractility‐maintaining mechanisms. However, sustained upregulation of SRF induces four‐chamber dilation, pathological hypertrophy, and interstitial fibrosis as it is a modulator of fetal genes like *Nppa* and *Acta1* (Frey & Olson, 2003; Miano, 2010). Therefore, it remains to be elucidated whether an induction of the *Srf* gene contributes to pathological cardiac phenotypes under chronic SB conditions. Overall, the increased expression of markers related to cell proliferation and cardiac hypertrophy point to a shift in cardiac structural balance and support the idea that TS mice are undergoing cardiac adaptation.

Pathological cardiac hypertrophy, as indicated by significantly increased TGF‐β levels, goes along with abnormal Ca^2+^ handling (Oldfield et al., 2019). In this context, connexins play a key role, as they control proper impulse propagation throughout the myocardium (Ehrlich et al., 2008). Heterogeneity in connexin 43 (encoded by the gene *Gja1*) distribution, found in pathologically hypertrophied rat ventricle, leads to uneven contraction. (Severs, 2001) Alterations of *Gja1* have been associated with abnormal impulse propagation and a steep increase in arrhythmia susceptibility (Danik et al., 2004; Severs, 2001). Our model presents an increase in expression of *Gja1*, which correlates with the results published by Moffit et al., underscoring that our results support the finding that tail suspension, and thus SB can cause pathological cardiac remodeling (Moffitt et al., 2013). Aquaporins (AQPs) are transmembrane channels that lead to cardiac irregularities when deficient in the heart (Verkerk et al., 2019). Increased expressions of *Aqp1* in isolated murine CM lead to hypertrophy, and low expressions, as found in our TS mice, are considered cardioprotective (Montiel et al., 2020). Noteworthy, other markers for Ca^2+^ handling and electrophysiological coupling, (i.e., *Atp2a2*, *Slc8a1*, and *Aqp7*) measured in this research remained unchanged.

In conclusion, a short period of 14 days of tail suspension leads to certain cardiac adaptation. It is important to mention that our structural markers point to hypertrophy and our Ca^2+^‐handling markers are altered. This partly confirms previous reports, although tail suspension‐induced cardiac phenotypes cover a wide spectrum. From the literature, it appears that four to 8 weeks of physical inactivity induced by tail suspension leads to cardiac malfunction, remodeling, and atrophy that is challenging recuperated after rest (Pogodina et al., 2004; Zhong et al., 2016). Our results show that even a short period of 2 weeks of physical inactivity induces cardiac alterations at the molecular level that can be cover both benign and hostile spectra linked with maladaptive hypertrophy. Therefore, our data shed new light on early phases of cardiac remodeling during physical inactivity conditions. Nonetheless, additional knowledge has to be gained in future studies to unravel the important question of how the heart adapts to inactivity phases during the important early phase.

## 5 STUDY LIMITATIONS

This study provides evidence of changes in metabolic, structural, and electrophysiological markers that occurred in the mouse heart upon after 2 weeks of tail suspension. Nevertheless, we are aware that our research has limitations that we would like to address. First, our method of dissecting the hearts prevents us from studying individual chambers which could yield a better understanding of the structural and electrophysiological changes on organ level. However, our aim is to discern the molecular changes that happen in the muscle rather than individual anatomical chambers, hence the use of whole heart for this analysis. Second, we cannot provide data on functional output of the hearts in vivo and ex vivo, as well as the lack of heart mass measurements. While functional outputs have been previously investigated in models similar to ours, the absence of such functional data in our study presents structural and electrophysiological level. Third, in this study only male mice were used. Our research, while not focused on differences on responses given by biological sex, is not inconceivable that the results are missing relevant information due to this. Given previously published data, we know that under pressure or volume overload, female sex is associated with lower disease burden (Tong et al., 2019). We suggest that future studies include female mice to fill this gap of knowledge. Lastly, due to technical difficulties and limited protein sample, we were not able to corroborate our results of the autophagy markers examine further the mTOR protein synthesis pathway.

## AUTHORS' CONTRIBUTIONS

Project design: FS; performing experiments: AVRG, MV, AP; contribution of reagents: FS; data analysis: AVRG, MV, FS; manuscript and figure drafting: AVRG, FS; final approval of manuscript: AVRG, MV, AP, FS.

## CONFLICT OF INTEREST

The authors declare no conflicts of interests.

## ETHICS STATEMENT

This project was approved by Animal Ethics Committee of the KU Leuven (P110/2018).

## Supporting information


Appendix S1.
