# Supplementary material for: Physical inactivity by tail suspension alters markers of metabolism, structure, and autophagy of the mouse heart
Source: Physiol Rep. 2023 Jan 25;11(2):e15574. doi: 10.14814/phy2.15574 (PMC9875748; doi:10.14814/phy2.15574)
Supplement: Supplementary file 1 — Appendix S1. [file PHY2-11-e15574-s001.pdf]

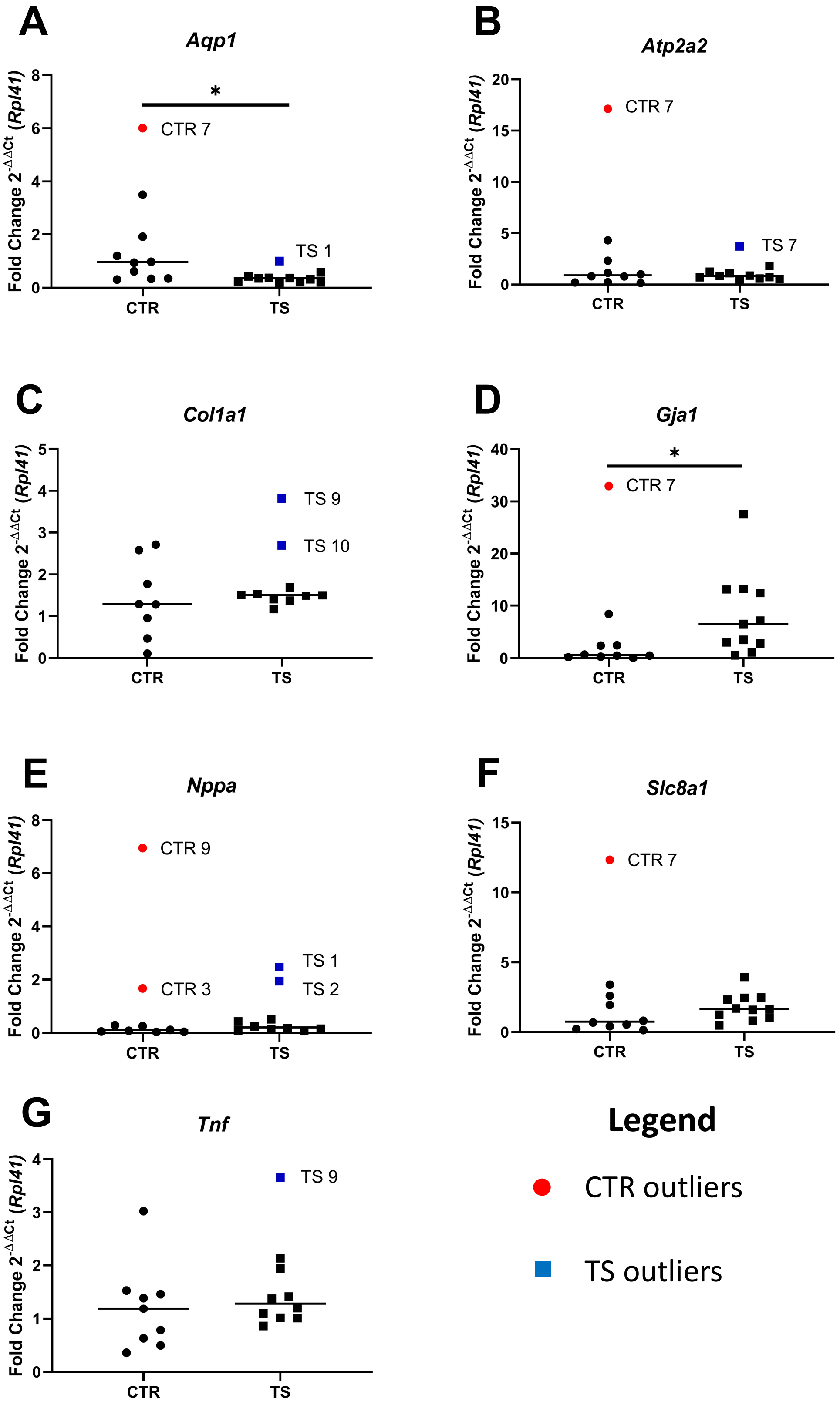
A
Aqp1
Fold Change 2^-ΔΔCt (Rpl41)
*
CTR 7
TS 1
CTR
TS
B
Atp2a2
Fold Change 2^-ΔΔCt (Rpl41)
CTR 7
TS 7
CTR
TS
C
Col1a1
Fold Change 2^-ΔΔCt (Rpl41)
TS 9
TS 10
CTR
TS
D
Gja1
Fold Change 2^-ΔΔCt (Rpl41)
*
CTR 7
CTR
TS
E
Nppa
Fold Change 2^-ΔΔCt (Rpl41)
CTR 9
CTR 3
TS 1
TS 2
CTR
TS
F
Slc8a1
Fold Change 2^-ΔΔCt (Rpl41)
CTR 7
CTR
TS
G
Tnf
Fold Change 2^-ΔΔCt (Rpl41)
TS 9
CTR
TS
Legend
CTR outliers
TS outliers

Figure 1.S.

Table 1.S. Descriptive data of gene markers presenting outliers

Gene	BEFORE REMOVING OUTLIERS									
	CONTROL					TAIL SUSPENSION				
	x	Avg	SD	SEM	p-value	x	Avg	SD	SEM	p-value
Aqp1	10	1.616	1.823	0.5766	0.0197	11	0.3884	0.2343	0.0706	0.0197
Atp2a	10	2.809	5.179	1.638	0.9177	11	1.151	0.9286	0.28	0.9177
Col1a1	8	1.394	0.9262	0.3275	0.2743	10	1.816	0.8153	0.2578	0.2743
Gja1	10	4.854	10.17	3.217	0.0242	11	8.286	7.946	2.396	0.0242
Nppa	9	1.055	2.268	0.7562	0.3562	10	0.6243	0.8582	0.2714	0.3562
Slc8a1	10	2.31	3.682	1.164	0.3494	11	1.79	0.9609	0.2897	0.3494
Tnf	9	1.208	0.8083	0.2694	0.3562	10	1.572	0.8377	0.2649	0.3562

Gene	AFTER REMOVING OUTLIERS									
	CONTROL					TAIL SUSPENSION				
	x	Avg	SD	SEM	p-value	x	Avg	SD	SEM	p-value
Aqp1	9	1.128	1.031	0.3436	0.0172	10	0.327	0.1219	0.0385	0.0172
Atp2a	9	1.221	1.337	0.4455	>0,9999	10	0.8957	0.4035	0.1276	>0,9999
Col1a1	8	1.394	0.9262	0.3275	0.8549	8	1.456	0.1516	0.0536	0.8549
Gja1	9	1.737	2.665	0.8885	0.0031	11	8.286	7.946	2.396	0.0031
Nppa	7	0.1258	0.1059	0.04	0.1818	8	0.2274	0.1622	0.0573	0.1818
Slc8a1	9	1.198	1.155	0.3851	0.2269	11	1.79	0.9609	0.2897	0.2269
Tnf	9	1.208	0.8083	0.2694	0.6712	9	1.341	0.4369	0.1456	0.6712

Table 1.S.

Table 2.S. Bodyweights from control and tail suspension mice

Animal	Bodyweights	
	CTR	TS
**1**	26.3	23.9
**2**	29.4	24
**3**	25.4	23.7
**4**	25.5	20.6
**5**	25.2	24.6
**6**	23.9	21.7
**7**	25.6	21.3
**8**	27.6	17.7
**9**	25	20.6
**10**	26	24.7
**11**		23.4

Table 2.S.
